# Impact of healthcare-associated infections within 7-days of acute stroke on health outcomes and risk of care-dependency: a multi-centre registry-based cohort study

**DOI:** 10.1007/s11739-024-03543-5

**Published:** 2024-03-22

**Authors:** David Fluck, Christopher H. Fry, Jonathan Robin, Brendan Affley, Puneet Kakar, Pankaj Sharma, Thang S. Han

**Affiliations:** 1 Department of Cardiology, Ashford & St Peter’s NHS Foundation Trust, Chertsey, GU9 0PZ UK; 2 School of Physiology, Pharmacology and Neuroscience, University of Bristol, Bristol, BS8 1TD UK; 3 Department of Acute Medicine, Ashford and St Peter’s NHS Foundation Trust, Chertsey, GU9 0PZ UK; 4 Department of Stroke, Ashford and St Peter’s NHS Foundation Trust, Chertsey, GU9 0PZ UK; 5 Department of Stroke, Epsom and St Helier University Hospitals, Epsom, KT18 7EG UK; 6 Department of Clinical Neuroscience, Imperial College Healthcare NHS Trust, London, W6 8RF UK; 7 Institute of Cardiovascular Research, Royal Holloway University of London, Egham, TW20 0EX UK; 8 Department of Endocrinology, Ashford and St Peter’s NHS Foundation Trust, Chertsey, GU9 0PZ UK

**Keywords:** Urinary tract infection, Pneumonia, Mortality, Hospital length of stay, Healthcare burden

## Abstract

Healthcare-associated infections (HCAIs) in patients admitted with acute conditions remain a major challenge to healthcare services. Here, we assessed the impact of HCAIs acquired within 7-days of acute stroke on indicators of care-quality outcomes and dependency. Data were prospectively collected (2014–2016) from the Sentinel Stroke National Audit Programme for 3309 patients (mean age = 76.2 yr, SD = 13.5) admitted to four UK hyperacute stroke units (HASU). Associations between variables were assessed by multivariable logistic regression (odds ratios, 95% confidence intervals), adjusted for age, sex, co-morbidities, pre-stroke disability, swallow screening, stroke type and severity. Within 7-days of admission, urinary tract infection (UTI) and pneumonia occurred in 7.6% and 11.3% of patients. Female (UTI only), older age, underlying hypertension, atrial fibrillation, previous stroke, pre-stroke disability, intracranial haemorrhage, severe stroke, and delay in swallow screening (pneumonia only) were independent risk factors of UTI and pneumonia. Compared to patients without UTI or pneumonia, those with either or both of these HCAIs were more likely to have prolonged stay (> 14-days) on HASU: 5.1 (3.8–6.8); high risk of malnutrition: 3.6 (2.9–4.5); palliative care: 4.5 (3.4–6.1); in-hospital mortality: 4.8 (3.8–6.2); disability at discharge: 7.5 (5.9–9.7); activity of daily living support: 1.6 (1.2–2.2); and discharge to care-home: 2.3 (1.6–3.3). In conclusion, HCAIs acquired within 7-days of an acute stroke led to prolonged hospitalisation, adverse health consequences and risk of care-dependency. These findings provide valuable information for timely intervention to reduce HCAIs, and minimising subsequent adverse outcomes.

## Introduction

Healthcare-associated (nosocomial) infections (HCAIs) are those that are acquired in a healthcare facility while patients are receiving treatment for another condition [1]. HCAIs remain a constant challenge to healthcare services [2] and have further been exacerbated by coronavirus disease (COVID)-19 [3]. Patients admitted with an acute stroke represent one of the highest risk groups for HCAIs because of their older age, multiple underlying health conditions [4], weakness and hyperacute cognitive stroke syndromes with distressing symptoms such as disorientation and delirium [5]. Brain-induced immunosuppression associated with stroke has also been implicated in the development of infections such as pneumonia for such patients [6, 7]. Patients with an acute stroke are highly susceptible to two major HCAIs: i) urinary tract infections (UTIs), associated with lower urinary tract dysfunction and which often require catheterisation [7, 8], and ii) pneumonia due to dysphagia [9]. Despite advances in stroke management, UTIs and pneumonia remain highly prevalent [7, 9].

Although studies have demonstrated that HCAIs are associated with a greater risk of death [10], several key indicators of the burden of disease and dependency, such as disability, malnutrition and care-support, have not been well-documented. Furthermore, the timing of HCAIs onset has often been poorly defined or they develop at the later stages of hospitalisation, thus making it difficult to interpret cause-and-effect relationships between HCAIs and outcome measures. In this study, we examined the impact of HCAIs acquired within 7-days of admission for an acute stroke on indicators of healthcare quality outcome and care-dependency, including: length of stay (LOS) on hyperacute stroke units (HASU); risk of malnutrition; requirement for palliative care; in-patient mortality; disability; as well as care-support on discharge, with a comprehensive adjustment for important confounding factors.

## Methods

### Study design, participants and setting

This study was part of the Sentinel Stroke National Audit Programme (SSNAP) [11]. We prospectively collected data from 3309 patients with an acute stroke who were consecutively admitted to four HASU in the south of England, from January 2014 to February 2016 [12].

### Socio-demographic factors and medical history

Socio-demographic factors were documented in detail by the stroke team, including: age at onset of stroke; sex; and co-morbidities including congestive heart failure; atrial fibrillation; hypertension; diabetes mellitus; and a history of previous stroke [11, 12].

### Diagnosis and severity of acute stroke

Diagnosis of stroke was based on clinical presentation and neuroimaging [11, 12] and classified as ischaemic stroke or intracranial haemorrhage. The severity of stroke symptoms at arrival was based on the National Institutes of Health for Stroke Scale (NIHSS), ranging from no symptoms (minimum NIHSS score = 0) to severe stroke symptoms (maximum NIHSS score = 42) [11, 12].

### Swallow screening and nutritional status

The target for conducting swallow screening was within 4-h of stroke diagnosis [11]. Oral fluid, food or medications were allowed if the patient had no risk of aspiration. Those with high risk of malnutrition were diagnosed according to the Malnutrition Universal Screening Test protocol [13].

### Healthcare-associated infections

Both UTI and pneumonia were diagnosed in the first 7-days following the initial admission for stroke. UTI was defined as patients who had a positive urine culture or were clinically treated, and newly acquired pneumonia was diagnosed on the basis of clinical examination and chest X-ray and treated with antibiotics [11, 12].

### Disability, mortality, palliative care, and care-support at discharge

Disability before the occurrence of stroke as well as at discharge was evaluated by the modified Rankin Scale (mRS). The mRS scores range from 0–6, with a higher score indicating a greater severity (mRS score = 6 indicates death) [14]. Mortality and palliative care at discharge were documented to reflect poor outcomes [11]. The level of care-support was planned for patients on discharge included help for activity of daily living (ADL), and discharge to a care-home [11, 12].

### Categorisation of variables

Moderately-severe to severe disability was defined as an mRS score ≥ 4. Severity of stroke was classified as: “no stroke symptoms” (NIHSS score = 0), “minor stroke” (NIHSS score = 1–4), “moderate stroke” (NIHSS score = 5–15), “moderate to severe stroke” (NIHSS score = 16–20), and “severe stroke” (NIHSS score = 21–42). Prolonged LOS was defined as those who spent longer than 14 days on HASU. Swallow screening status was categorised into groups: screening performed within 4 h, 4–72 h, and > 72 h of stroke diagnosis [11, 12]. Age stratification was based on three groups: < 70, 70–79, and ≥ 80 years.

### Statistical analysis

Kruskal–Wallis tests were conducted to test HCAIs in non-parametric data (LOS on HASU). Multivariable logistic regression was conducted to examine the association between HCAIs (patients without HCAIs as reference) and healthcare outcomes and dependency (LOS in hospital > 14 days; palliative care decision by discharge; in-patient mortality; mRS ≥ 4 at discharge; risk of malnutrition; ADL support and discharge to care-home), with adjustment for risk factors (age; sex; co-morbidities; mRS scores; type of stroke; NIHSS scores; and time taken for swallow screening). The results were expressed as odds ratios (OR) and 95% confidence intervals (CI). The goodness-of-fit for logistic regression was assessed by Hosmer–Lemeshow tests. Analyses were performed using SPSS Statistics for Windows, v.28.0 (IBM Corp., Armonk, NY, USA).

## Results

### Patient characteristics

A total of 3309 patients (1656 men and 1653 women) were studied, with men younger (mean ± SD) at of onset of stroke (73.1 ± 13.2 years) than women (79.3 ± 13.0 years). Hypertension represented the highest proportion of patients with underlying risk factors for stroke (52.3%), followed by history of previous stroke (23.1%), atrial fibrillation (20.1%), diabetes (16.0%), and congestive heart failure 5.9%). There were 5.5% of patients with pre-stroke disabilities (mRS score ≥ 4). Most patients were diagnosed with ischaemic stroke (83.3%, with the remainder mostly as intracranial haemorrhage (15.7%), and 7.7% had moderate-to-severe stroke (NIHSS score = 16–20) and 6.9% had severe stroke (NIHSS score > 20) on arrival. Within 7-days of admission, UTI and pneumonia occurred in 7.6% and 11.3% of patients, respectively. There were 33.9% of patients staying on HASUs > 14 days, 25.8% at risk of malnutrition, 14.5% in-patient deaths and 7.6% with a decision made for palliative care on discharge. At discharge, 29.9% of patients had disabilities (mRS score ≥ 4), 20.4% required ADL support and 5.3% required new care-home discharge (Table 1).

**Table 1:** Distribution of 3309 patients, 1656 men and 1653 women admitted with stroke to hospitals in Surrey between January 2014 and February 2016

	n	Proportion (%)
On admission		
Men: Women	1656: 1653	50.0: 50.0
Comorbidities		
Pre-stroke disability (mRS score ≥ 4)	181	5.5
Congestive heart failure	194	5.9
Atrial fibrillation	666	20.1
Previous stroke	766	23.1
Hypertension	1729	52.3
Diabetes	531	16.0
Type of stroke on arrival		
Ischaemic stroke: intracranial haemorrhage: unspecified	2758: 518: 33	83.3: 15.7: 1.0
Severity of stroke on arrival		
No stroke symptoms (NIHSS score = 0)	444	13.4
Minor stroke (NIHSS score = 1–4)	1263	38.2
Moderate stroke (NIHSS score = 5–15)	1120	33.8
Moderate to severe stroke (NIHSS score = 16–20)	255	7.7
Severe stroke (NIHSS score = 21–42)	227	6.9
Healthcare-associated infections		
Urinary tract infection within 7-days	243	7.6
Pneumonia within 7-days	358	11.3
Urinary tract infection and/or pneumonia within 7-days	478	15.0
Stroke outcomes during hospitalisation		
Staying on hyperacute stroke unit > 14 days	892	33.9
Risk of malnutrition	853	25.8
Palliative care decision by discharge	253	7.6
In-patient mortality	480	14.5
Disability on discharge (mRS score ≥ 4)	989	29.9
Activities of daily living support	544	20.4
New care-home discharge	177	5.3

mRS, modified Rankin Scale; NIHSS, National Institutes of Health Stroke Scale

Table 2 shows the main features of patients with different HCAI status. Compared to those without either UTI or pneumonia, proportionally there were: more women; older patients (≥ 80 years); greater prestroke disabilities and comorbidities; more severe stroke, and poorer outcomes. These proportions were generally higher amongst those with pneumonia only than those with UTI only, and further increased (except for congestive heart failure, risk of malnutrition and new discharge to care homes) in those with both HCAIs.

**Table 2:** Features in stroke patients according to of healthcare-associated infection status

	healthcare-associated infection status (%)				
	None	UTI only	Pneumonia only	UTI and pneumonia	χ^2^ test for group differences (*P*)
Women	47.9	76.7	52.3	63.4	< 0.001
Age ≥ 80 yrears	44.6	65.8	68.5	70.7	< 0.001
Comorbidities					
Pre-stroke disability (mRS score ≥ 4)	4.3	11.7	8.5	22.0	< 0.001
Congestive heart failure	5.7	7.5	6.8	6.5	0.417
Atrial fibrillation	17.8	27.5	33.6	42.3	< 0.001
Previous stroke	22.1	27.5	28.9	28.5	0.004
Hypertension	50.3	65.0	60.9	63.4	< 0.001
Diabetes	15.7	19.2	17.9	21.1	0.063
Intracranial haemorrhage	14.5	17.5	20.2	25.2	< 0.001
Severity of stroke on arrival					
No stroke symptoms (NIHSS score = 0)	15.1	3.3	3.4	2.4	< 0.001
Minor stroke (NIHSS score = 1–4)	42.2	34.2	16.2	13.0	
Moderate stroke (NIHSS score = 5–15)	32.2	46.7	39.6	40.7	
Moderate to severe stroke (NIHSS score = 16–20)	5.7	7.5	22.6	15.4	
Severe stroke (NIHSS score = 21–42)	4.8	8.3	18.3	28.5	
Stroke outcomes during hospitalisation					
Staying on hyperacute stroke units > 14 days	29.6	62.4	80.8	81.8	< 0.001
Risk of malnutrition	21.9	37.8	71.4	49.6	< 0.001
Palliative care decision by discharge	5.4	4.3	32.9	54.3	< 0.001
In-patient mortality	9.8	8.3	47.7	73.2	< 0.001
Disability on discharge (mRS score ≥ 4)	23.1	50.0	81.7	89.4	< 0.001
Activities of daily living support	18.9	35.2	30.2	50.0	< 0.001
New care-home discharge	4.3	15.8	13.2	8.1	< 0.001

mRS, modified Rankin Scale; NIHSS, National Institutes of Health Stroke Scale; UTI, urinary tract infection

### Outcome measures

**Fig. 1:**
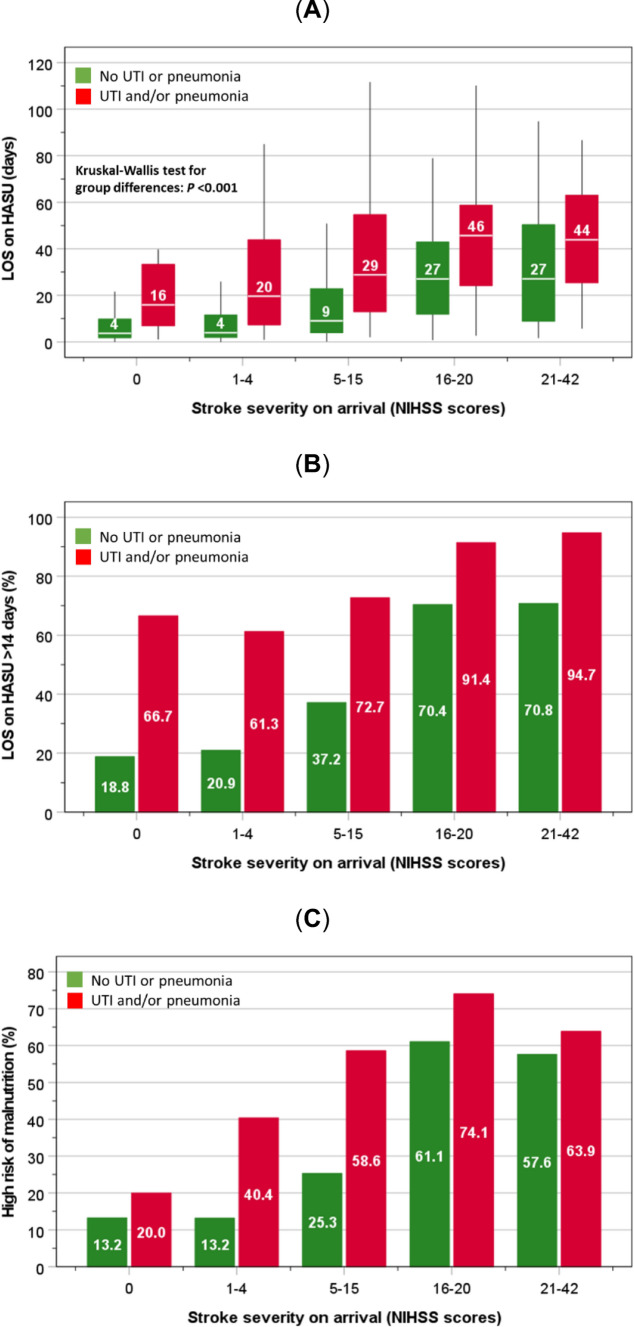
Number of days spent on HASU (**A**), and proportions of patients staying > 14 days on HASU (**B**) and high risk of malnutrition (**C**), according to HCAI status and stroke severity. Boxes in part **A** represent median and interquartile ranges and whiskers represent the 5th and 95th percentiles

**Fig. 2:**
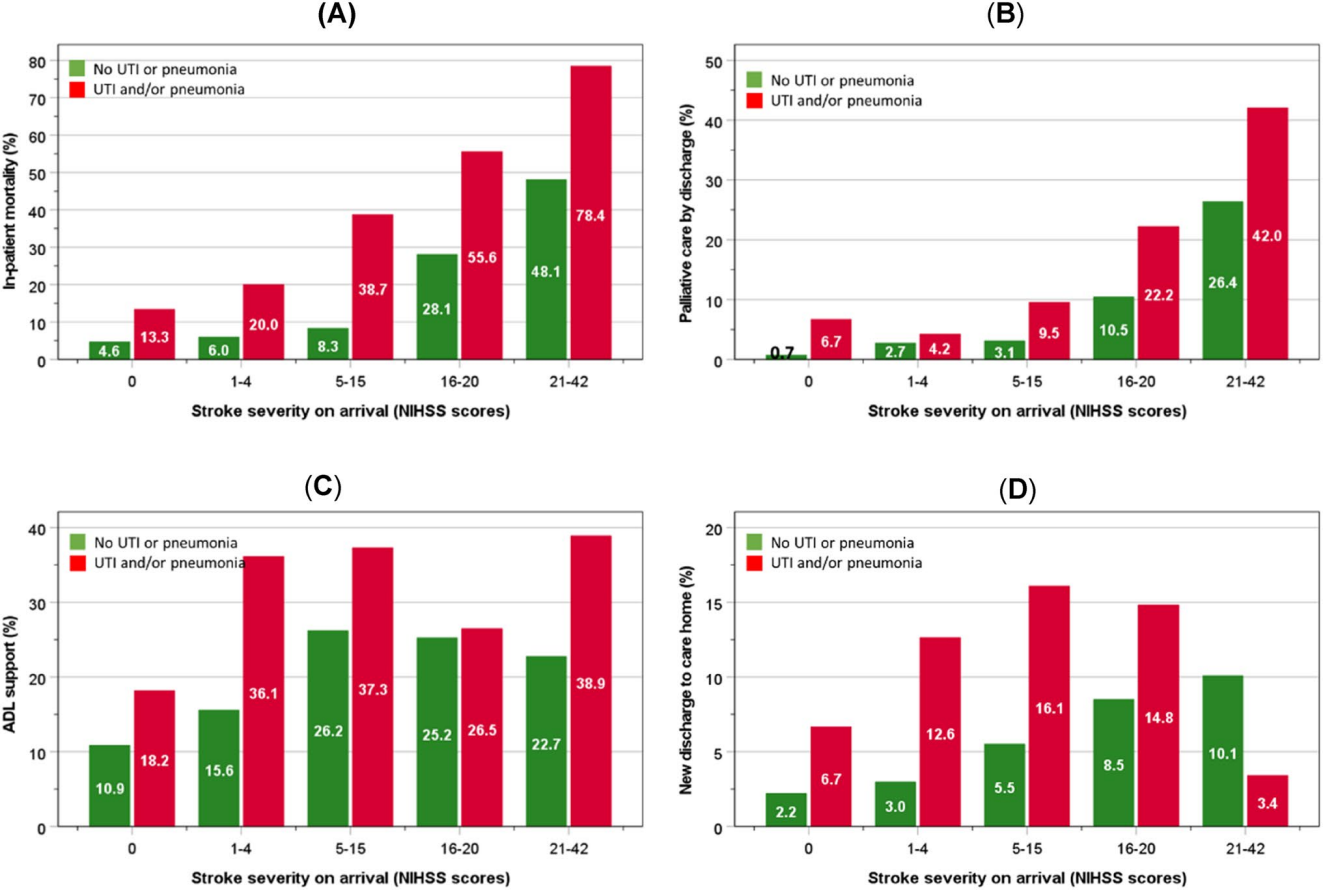
Proportions of patients who died (**A**), required palliative care by discharge (**B**), ADL support at discharge (**C**), and new discharge to home-care (**D**), according to HCAI status by stroke severity

For any given severity of stroke, the LOS on HASU was longer (Fig. 1A), whilst there were increasingly higher proportions of patients spending > 14 days on HASU and a high risk of malnutrition (Fig. 1B, C); in-patient mortality; palliative care; ADL support; and new discharge to a care-home (except for the group with most severe strokes) (Fig. 2A–C).

Event rates, unadjusted and adjusted OR and 95%CI are shown in Table 3. After adjusting for potential confounding factors (age; sex; co-morbidities; pre-stroke disabilities; swallow screening; stroke type and severity) and compared to patients without UTI, patients with UTI were more likely to have (OR, 95%CI) prolonged stay (> 14-days) on HASU: 3.3 (2.4–4.8); high risk of malnutrition: 1.5 (1.1–2.0); palliative care decision: 2.5 (1.7–3.7); in-hospital mortality: 3.0 (2.2–4.1); disability at discharge: 3.9 (2.8–5.4); ADL support: 1.8 (1.2–2.6); and discharge to care-home: 1.9 (1.2–3.0). Compared to patients without pneumonia, those with pneumonia were also more likely to have prolonged stay on HASU: 7.4 (4.9–11.4); high risk of malnutrition: 4.6 (3.6–5.9); palliative care decision by discharge: 7.7 (5.6–10.6); in-hospital mortality: 8.2 (6.2–10.8); disability at discharge: 12.5 (9.0–17.2); ADL support: 1.6 (1.1–2.4); and discharge to care-home: 1.9 (1.3–2.8).

**Table 3:** Logistic regression to estimate odd ratios of outcomes associated with urinary tract infection and with pneumonia acquired within 7-days of admission for an acute stroke

Urinary tract infection within 7-days of admission^†^	Unadjusted				Adjusted*		
	Event rates (%)	OR	95%CI	*P*	OR	95%CI	*P*
Length of stay on hyperacute stroke unit > 14 days	10.7	4.29	2.99–6.14	< 0.001	3.32	2.28–4.84	< 0.001
Risk of malnutrition	12.3	2.23	1.71–2.92	< 0.001	1.46	1.09–1.95	0.011
Palliative care decision by discharge	17.1	3.73	2.58–5.40	< 0.001	2.47	1.65–3.70	< 0.001
In-patient mortality	21.0	4.75	3.60–6.27	< 0.001	2.97	2.15–4.11	< 0.001
Disability on discharge (mRS score ≥ 4)	17.2	6.06	4.55–8.06	< 0.001	3.91	2.82–5.42	< 0.001
Activities of daily living support	9.7	2.62	1.83–3.74	< 0.001	1.78	1.22–2.60	0.003
New care home discharge	16.4	2.56	1.68–3.89	< 0.001	1.91	1.23–2.98	0.004
Pneumonia within 7-days of admission^‡^							
Length of stay on hyperacute stroke unit > 14 days	13.9	9.51	6.29–14.37	< 0.001	7.43	4.85–11.39	< 0.001
Risk of malnutrition	26.4	6.08	4.81–7.69	< 0.001	4.56	3.55–5.86	< 0.001
Palliative care decision by discharge	42.9	10.98	8.20–14.70	< 0.001	7.66	5.55–10.58	< 0.001
In-patient mortality	42.4	12.04	9.44–15.36	< 0.001	8.21	6.24–10.81	< 0.001
Disability on discharge (mRS score ≥ 4)	30.7	16.89	12.54–22.75	< 0.001	12.46	9.04–17.18	< 0.001
Activities of daily living support	9.4	2.16	1.52–3.07	< 0.001	1.63	1.12–2.38	0.011
New care-home discharge	23.2	2.55	1.77–3.69	< 0.001	1.87	1.26–2.77	0.002

mRS, modified Rankin Scale, *Adjusted for age; sex; co-morbidities; mRS scores; type of stroke; National Institutes of Health Stroke Scale scores; and time taken for swallow screening; reference groups: ^**†**^without UTI or ^**‡**^without pneumonia

**Fig. 3:**
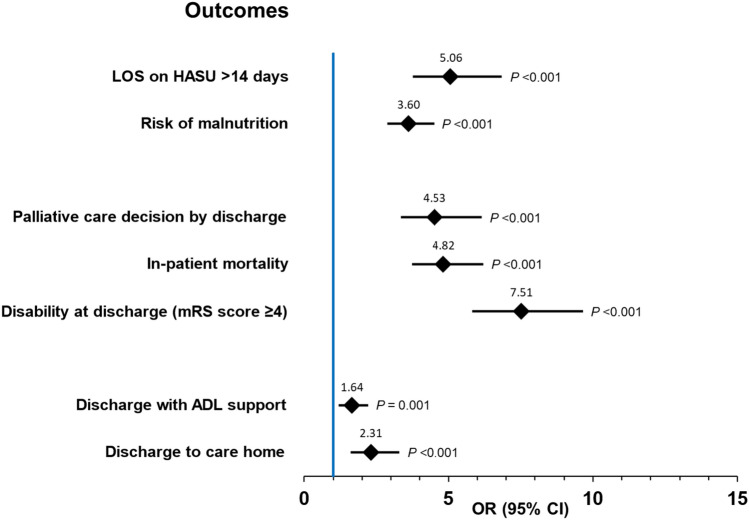
Outcomes for patients with HCAIs acquired within 7-days of admission for acute stroke (reference group: without UTI or pneumonia). Data were adjusted for age, sex, comorbidities, pre-stroke disability, severity of stroke on arrival and delay in swallow screening

Further analysis was conducted comparing those without UTI or pneumonia. Patients with UTI and/or pneumonia had greater adjusted risk for having prolonged length of stay on HASU: 5.1 (3.8–6.8); high risk of malnutrition: 3.6 (2.9–4.5); palliative care decision: 4.5 (3.4–6.1); in-hospital mortality: 4.8 (3.8–6.2); disability at discharge: 7.5 (5.9–9.7); ADL support: 1.6 (1.2–2.2); and discharge to care-home: 2.3 (1.6–3.3) (Fig. 3).

## Discussion

In summary, for acute stroke patients hospital-acquired UTI increased the risk of prolonged stay on HASUs, death, palliative care, or disability by 2.5–4 times, and a greater risk of malnutrition, ADL support or discharge to care-homes by 1.5–2 times. Pneumonia increased the risk of prolonged stay on HASUs, death, palliation, or disability by 7.4–12.5 times, and the risk of malnutrition, ADL support or discharge to care-homes by 1.6–4.6 times. Furthermore, these HCAIs exerted their effects on outcomes at every level of stroke severity.

The influence of these HCAIs on an increased LOS is consistent with previous studies of stroke [15–17] and other medical conditions [18]. A prolonged hospital stay induces loss of bodily functions [19] and predisposes patients to infections, risk of malnutrition and death [20]. This vicious cycle also increases healthcare costs. A study in Scotland estimated that 58,010 hospital bed-days were lost to HCAIs, at an annual cost of £46.4 million, which extrapolated to £774 million overall in the UK [21]. With another study in England, 52,085 UTI and 7,529 bloodstream infections were associated with bladder catheterisation, of which 38,084 and 2,524 respectively were HCAIs. Catheter-associated UTI (CAUTI) incurred 45,717 excess bed-days, 1,467 deaths and 10,471 lost quality-adjusted life-years (QALYs). Estimated total direct hospital costs amounted to £54.4 million, with an additional £209.4 million in economic value of lost QALYs [22].

With acute stroke patients, associations of HCAIs with increased risk of death [8, 16, 17, 23] and disability [15, 17, 23, 24] are well documented and consistent with this study, but little information is available for palliative care. Overall they indicate the seriousness of HCAIs and the need for early identification of those at greatest risk as they continue to have an impact on disability [17] and mortality [25] for many years.

The burden of stroke is enormous globally [26] and we found acute stroke patients with HCAIs were more likely to require ADL support and care-homes. This area is poorly studied, but such patients impose high burdens of disease to caregivers and healthcare services [27]. Moreover, the total health and social care cost for acute stroke patients in the UK (except Scotland) is about £3.60 billion a year in the first 5-years after admission, with an average cost of £46,039 per patient [28]. However, there is a lack of information on the impact of HCAIs on the overall cost of post-stroke care.

Direct causal links between HCAIs and subsequent outcomes cannot be established by this study. However, unique features include: (i) a sequential timeline of variables of interest: starting from underlying risk factors occurring prior to development of HCAIs, followed by HCAIs acquired during the early phase of admission, and subsequent outcome measures that developed after the occurrence of HCAIs; (ii) risk factors were independently associated with HCAIs, and (iii) these risk (confounding) factors, which could influence the association between HCAIs and outcomes, were accounted for. Poor outcomes were further accentuated by the presence of HCAIs, at any stroke severity. To recognise HCAIs at a specific time of hospitalisation, especially during the initial phase of acute stroke is therefore crucial, with respect to timely treatment and evaluation of the possible causal links between HCAIs and outcomes.

Although HCAIs are particularly detrimental to patients admitted with an acute stroke, they are widespread across medical and surgical patients, imposing a huge burden of disease on healthcare services. Amongst 13.8 million adult patients admitted to National Health Service (NHS) hospitals in England in 2016–2017, 653,000 (4.73%) developed HCAIs, of whom 22,800 (3.49%) died as a result [29]. For patients admitted with acute general medical conditions, bloodstream, respiratory and urinary tract infections are amongst the most common HCAIs across high-income countries [30, 31], with older age and underlying health conditions being the major risk factors [32]. HCAIs are associated with increased mortality and morbidity [33], LOS in hospital [18] and burden of disease [34].

Indwelling urinary catheters are used frequently in older adults admitted to hospital, but introduce CAUTI. In a 2019 study of 5,203,496 patients admitted for acute medical conditions, 19.2% were catheterised, of whom 3.8% developed CAUTI in hospital [22]. Stroke patients are at a 3.5-fold greater risk of UTI than acute medical patients [35], possibly exacerbated by catheterisation [7]. The rates of CAUTI in this study are not known as the SSNAP protocol does not record information on the use of indwelling urinary catheters. However, Stott et al.reported that amongst stroke patients, catheters were used in 18% of those without and 63.1% with UTI [8], whilst another study found the risk of UTI amongst stroke patients was increased by 2.7-fold for those with a catheter placement compared to those without [36]. However, catheterisation rates in acute stroke patients were higher than those with acute medical conditions, but have been somewhat declining over time. In an earlier study of 3,756 stroke patients admitted to London hospitals in 1995–2011, 31.2% were catheterised [37], whilst a 2004–2005 study of 404 acute stroke patients in Scotland showed 25.7% had catheter insertion [8]. A 2006–2008 study of 2,893 Taiwanese stroke patients showed 25% received catheterisation [38] and a smaller 2013 study of 212 French stroke patients, reported 21.2% had urinary catheters inserted [39].

However, despite their widespread use, routine documentation of indwelling urinary catheters in stroke patients is lacking at a national level, including SSNAP [40, 41]. Thus, up-to-date progress on reducing CAUTI in stroke patients is unclear. Given the impact of UTI on poor outcomes, the use of indwelling urinary catheters should be documented routinely to monitor and minimise avoidable catheterisation, or reduce the duration of their use, and consequently improve the quality of patient care [42].

Early nutritional support is also crucial in stroke survival. Early identification of dysphagia allows timely delivery of parenteral or enteral nutritional support through a central venous line or a nasogastric tube respectively. Although these are routinely used in a clinical setting, the SSNAP protocol does not record the rates of nasogastric tube (NGT) insertion; therefore its risks and benefits could not be assessed. However, a systemic review of eleven articles (60–1088 patients) showed no clear evidence for an association between NGT placement and stroke-associated pneumonia [9]. Further prospective studies are therefore warranted to assess the risk of stroke-associated pneumonia from a NGT compared to other routes of nutritional support.

Summaries of UK stroke data between 2013 and 2023 [40, 41] showed that age, sex distribution and co-morbidities of acute stroke remain generally unchanged. However, certain aspects of stroke management have improved over time. National SSNAP data through 2013/14 to 2022/23 revealed that brain imaging within 1-h of arrival had increased from 42 to 57%, and access to specialist care for intracranial haemorrhage increased from 66 to 76%. This was linked to lower in-hospital mortality from 33 to 29%, whilst discharge to an Early Supported Discharge or Community Rehabilitation Team increased from 41 to 61%. However, an initial rise of patients receiving 45 min of occupational therapy, physiotherapy, speech and language therapy and psychology five days a week declined during the Coronavirus-19 pandemic [40, 41]. These changes over time may have some bearing on the data analyses from this study which collected data from 2014–2016. However our study examined the risk of HCAIs on health outcomes, as opposed to measuring prevalence or incidence. Therefore results were less temporally-dependent, especially when the data were fully adjusted for time-dependent confounding factors. Thus, in this study the relative risk of an outcome due to HCAIs would be expected to be similar to that at the present time.

Limitations to this study include the limited number of principal HCAIs (UTI and pneumonia) collected by the SSNAP protocol, and it would be important in future studies to document other HCAIs such as bloodstream infections and gastrointestinal complications. Although the study recruited patients locally, the data were from a relatively large cohort of patients admitted consecutively from one of the largest NHS regions in the UK, and data collection followed national SSNAP guidance, using standardised methods [11].

In conclusion, our study provided further insights into the impact of HCAIs on healthcare outcomes in acute stroke patients. Timely intervention is therefore necessary to reduce/prevent HCAIs.

## Data sharing statement

No additional data are available.

## Data Availability

Not applicable.

## Abbreviations

ADL: Activity of daily living
CAUTI: Catheter-associated urinary tract infection
CI: Confidence intervals
COVID: Coronavirus disease
HASU: Hyperacute stroke units
HCAIs: Healthcare-associated infections
LOS: Length of stay
mRS: Modified Rankin Scale
NGT: Nasogastric tube
NHS: National Health Service
NIHSS: National Institutes of Health for Stroke Scale
OR: Odds ratios
QALYs: Quality-adjusted life-years
SSNAP: Sentinel Stroke National Audit Programme
UTI: Urinary tract infection
